# Feasibility of generating structured motivational messages for tailored physical activity coaching

**DOI:** 10.3389/fdgth.2023.1215187

**Published:** 2023-09-12

**Authors:** Ramya P. Ghantasala, Nele Albers, Kristell M. Penfornis, Milon H. M. van Vliet, Willem-Paul Brinkman

**Affiliations:** ^1^ Department of Intelligent Systems, Delft University of Technology, Delft, Netherlands; ^2^ Unit Health Medical and Neuropsychology, Institute of Psychology, Leiden University, Leiden, Netherlands; ^3^ Department of Cardiology, Leiden University Medical Center, Leiden, Netherlands; ^4^ Public Health and Primary Care, Leiden University Medical Center, Leiden, Netherlands; ^5^ National eHealth Living Lab, Leiden University Medical Center, Leiden, Netherlands

**Keywords:** physical activity, behavior change, eHealth, tailoring, personalization, motivation, feedback, monitoring

## Abstract

Tailored motivational messages are helpful to motivate people in eHealth applications for increasing physical activity, but it is not sufficiently clear how such messages can be effectively generated in advance. We, therefore, put forward a theory-driven approach to generating tailored motivational messages for eHealth applications for behavior change, and we examine its feasibility by assessing how motivating the resulting messages are perceived. For this, we designed motivational messages with a specific structure that was based on an adaptation of an existing ontology for tailoring motivational messages in the context of physical activity. To obtain tailored messages, experts in health psychology and coaching successfully wrote messages with this structure for personas in scenarios that differed with regard to the persona’s mood, self-efficacy, and progress. Based on an experiment in which 60 participants each rated the perceived motivational impact of six generic and six tailored messages based on scenarios, we found credible support for our hypothesis that messages tailored to mood, self-efficacy, and progress are perceived as more motivating. A thematic analysis of people’s free-text responses about what they found motivating and demotivating about motivational messages further supports the use of tailored messages, as well as messages that are encouraging and empathetic, give feedback about people’s progress, and mention the benefits of physical activity. To aid future work on motivational messages, we make our motivational messages and corresponding scenarios publicly available.

## 1. Introduction

Tailored motivational messages have been shown to be useful in eHealth applications for behavior change such as ones for becoming more physically active (1–4). For example, providing users with actionable feedback (i.e., feedback that can be acted on) in combination with information about their progress has been found to increase intrinsic motivation as well as adherence (5). For somebody creating an eHealth application, this means that they need to find a way to generate motivational messages adjusted to each combination of values for the tailoring factors that might be relevant to consider, such as a person’s progress or motivation. Since it is likely not feasible for experts to write and send these messages in real time after the application has been deployed, the messages should be generated in advance.

A common approach to generating motivational messages used in current eHealth applications for behavior change is writing custom and hand-crafted messages without a specific structure (e.g., (6–10)). While this approach may lead to messages that are effective for the specific application at hand, it is difficult for other researchers to follow the same recipe for generating messages for a different behavior change application. This hinders the reuse, reproducibility, and generalizability of findings, especially when the messages are also not publicly shared (e.g., (6–8, 11, 12)).

To obtain structured motivational messages, both data-driven and theory-driven approaches have been employed. For example, Tielman et al. (13) asked experts to write messages for scenarios differing based on user trust and therapy progress in the context of post-traumatic stress disorder therapy. The sentences in the resulting messages were used to identify categories such as “compliment” and “give perspective,” and new messages were generated based on the probabilities of categories appearing for a certain combination of tailoring factors. The resulting messages thus do have a specific structure, but this structure is based on data from a specific domain and may hence not be applicable to other domains. An alternative to this data-driven approach is a theory-driven one. Thomas et al. (14), for example, proposed asking peers to write messages for healthy eating based on argumentation schemes (i.e., forms of argument). Because the resulting message structure is not tailored to a specific domain, it can be used in different behavior change domains, thus reducing the time needed for designing and validating messages for the new domain. However, limited work on such theory-driven approaches exists for generating motivational messages that are tailored.

Our goal was thus to develop and test the feasibility of a theory-driven approach to generating tailored motivational messages. We, therefore, asked experts in health psychology and coaching to write motivational messages with a specific structure derived from an ontology. To obtain tailored messages, these messages were written for personas in scenarios that differed with regard to the mood, self-efficacy, and progress of the persona in the context of becoming more physically active. A variety of tailoring factors for motivational as well as persuasive messages more generally has been examined in previous work, including both dynamic factors (e.g., progress (15), self-efficacy (16), states derived from the Capability-Opportunity-Motivation-Behavior (COM-B) model (17)) and more stable user characteristics (e.g., gender (18), need for cognition (19), physical activity identity (20)). In line with our goal, we chose three factors that have been shown to be relevant in previous work and not necessarily the most important factors. Progress was chosen to make progress feedback possible, which can help users keep a positive mindset about physical activity (15), increase users’ motivation to reach their goal behavior (21), and make messages more interesting (22) and thus more likely to be processed in detail (23) and with a persistent impact (24); mood and self-efficacy were primarily chosen because they can affect how messages and feedback, in particular, are processed. Specifically, mood has been shown to affect how a user processes messages (25) and feedback regarding behavior change (26, 27), and self-efficacy influences how a user absorbs message content (28) and is persuaded by differently framed messages for behavior change (16). Mood and self-efficacy can thus affect how effective different motivational messages are. Furthermore, mood and self-efficacy both influence physical activity and are influenced by it (26, 29, 30). This can be taken into account in motivational messages for physical activity if a user’s mood and self-efficacy are known.

To validate our resulting messages, we conducted an experiment in which 60 participants each rated the perceived motivational impact of six tailored and six generic messages based on scenarios. Our hypothesis was that the tailored messages are perceived as more motivating than the generic ones. To improve motivational messages in the future, we further qualitatively analyzed people’s free-text responses about what they find motivating and demotivating about motivational messages. Our results support the feasibility of theory-based generation and the use of messages tailored to mood, self-efficacy, and progress. To aid future work on motivational messages, we make the scenarios and resulting 60 tailored and 12 generic motivational messages publicly available (31).

## 2. Method

Our experiment was run from December 2021 to January 2022. The Human Research Ethics Committee of Delft University of Technology granted ethical approval for the research (Letter of Approval number: 1814) and the experiment was pre-registered on the Open Science Framework (OSF) (32).

### 2.1. Experimental design

The study was set up as a double-blind within-subjects experiment. The within-subjects factor was the type of message that participants rated for a hypothetical scenario. In the control condition, participants rated generic messages; in the experimental condition, they rated messages tailored to progress, mood, and self-efficacy. To mitigate order and learning effects, ABBA counterbalancing (33) was performed when assigning tailored or generic messages to participants. Furthermore, the progress, mood, and self-efficacy of the persona used in the hypothetical scenarios were counterbalanced with a modified Latin square.

### 2.2. Materials

The online crowdsourcing platform Prolific and the survey platform Qualtrics were used to recruit participants and host the questionnaires.

**Scenarios**. 30 hypothetical scenarios were designed to elicit motivational messages from experts. The scenarios were created by combining three levels of mood, two levels of self-efficacy, and five levels of progress (2×3×5=30). Each scenario had a persona with a specific mood, self-efficacy, and progress level of their physical activity program. The list of scenarios can be found in our online repository. Two independent coders labeled each scenario with a mood, self-efficacy, and progress level to evaluate whether each scenario had a distinguishable mood, self-efficacy, and progress level. We obtained substantial to almost perfect agreement (34) for mood (κ=0.73), self-efficacy (κ=0.73) and progress (κ=0.96).

**Figure 1:**
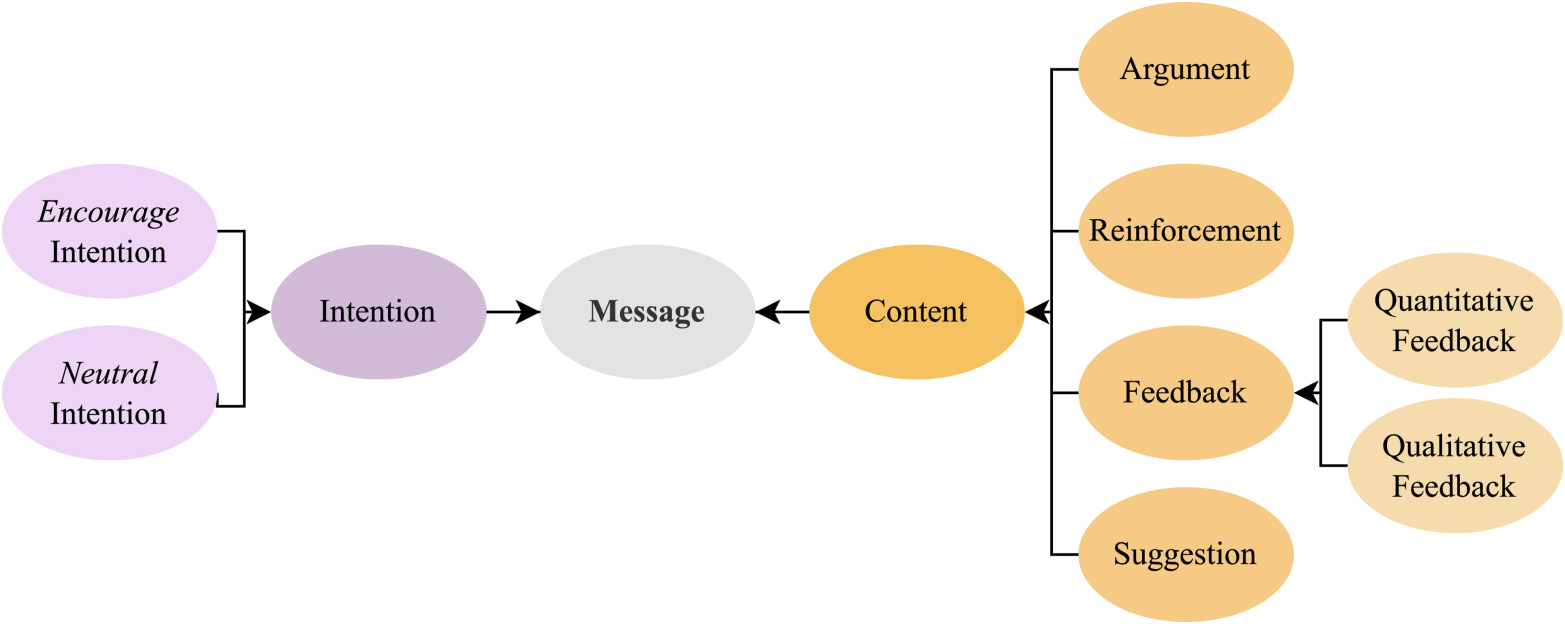
The ontology used for generating tailored motivational messages to improve physical activity. Based on the ontology developed by Op den Akker et al. (27), we have added the components of qualitative and quantitative feedback, while retaining the *encourage* and *neutral* intentions as well as the suggestion, argument, and reinforcement components.

**Figure 2:**
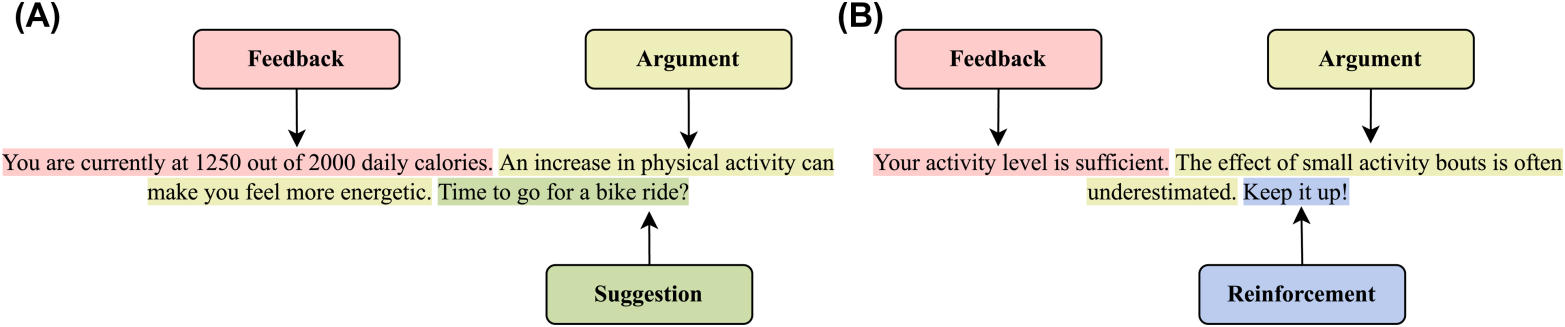
Message structure for messages with (**A**) *encourage* and (**B**) *neutral* intentions based on Op den Akker et al. (27). An *encourage* intention encourages users to do (more) physical activity, whereas a *neutral* intention acknowledges a user’s progress and informs them to keep up the good work. If a user is consistently achieving their goals, they need not be reminded frequently about the arguments to stay active, and hence only the feedback and reinforcement components can then be used for messages with *neutral* intentions.

**Motivational messages**. Using the framework by Op den Akker et al. (27) as inspiration, we created an ontology to model tailored motivational messages for physical activity coaching based on their intention (i.e., why the message is sent) and content (i.e., what is in the message) (Figure 1). The message structure (Figure 2) consists of the components feedback, argument, reinforcement, and suggestion following the model by Op den Akker et al. (27), which was created as a model of tailoring for real-time physical activity coaching applications based on a survey of the literature and of messages used in previous studies. Two health psychologists with experience in behavioral change coaching and developing eHealth applications for increasing physical activity were recruited from the network of the authors to write tailored motivational messages for the 30 hypothetical scenarios based on this ontology for a total of 60 messages. The psychologists were asked to consider the people in the scenarios as their clients or patients and write the messages as if they were motivating them to improve their physical activity levels. Along with the tailored messages, the psychologists were asked to write six generic messages each to motivate people to increase their physical activity regardless of their mood, self-efficacy, or progress. The psychologists were instructed to write all messages using the message structure depicted in Figure 2 if possible. All messages are provided in our online repository.

### 2.3. Measures

We tested our main hypothesis using the following measure:

**Perceived motivational impact of messages**. For each message, participants rated the perceived motivational impact on an 11-point scale from −5 (“Very demotivating” ) to 5 (“Very motivating”), adapted from the one by de Vries et al. (35). A higher value thus indicates a higher perceived motivational impact.

In addition, we collected the following measures for exploratory research:

**Motivating and demotivating factors of messages**. Participants were asked to provide free-text answers to the questions “How would a message motivate you to do physical activity?” and “How would a message demotivate you from doing physical activity?” The questions were adapted from the ones by Fukuoka et al. (36).

### 2.4. Participants

We conducted a power analysis for repeated measures, within-factors ANOVA with 6 repeated measurements per condition (i.e., 12 measurements in total), a small effect size (f=0.18) (37), an alpha of 0.05, and a power of 0.90 to obtain a target sample size for our Bayesian analysis. The result was a sample size of 30. This estimate was conservative, as Bayesian analyses require no more samples than the corresponding frequentist ones (38). We also decided to add an additional 30 participants for a total of 60 if we had sufficient funds left after the first 30 participants had completed the study. This was done to increase the power, as well as to get more responses for the qualitative analysis. The decision to include more participants was taken without looking at experimental data.

To be eligible, participants had to meet the following criteria:

1. have an approval rate of ≥95% on the crowdsourcing platform Prolific to obtain participants with reliable submissions (39),
2. speak English fluently, and
3. have at least 10 previous submissions on Prolific, to avoid low-effort or low-quality submissions (as recommended by Prolific to recruit active and committed users (40)).

60 participants completed the study out of a total of 121 who started it. If participants passed at least half of the four and two attention check questions added in the two sections of the questionnaire respectively (i.e., the pre-questionnaire and scenario-rating sections), gave informed consent, and passed the pre-screener validation about English fluency, they were approved and paid according to the minimum payment rules on Prolific (i.e., five pounds sterling per hour). Participants were informed that their responses to the questionnaire would not affect their payment in any way unless it violated the conditions mentioned above. This served to mitigate biases such as the ones mentioned by Draws et al. (41), specifically loss aversion and self-interest bias. Participant characteristics such as age and gender are provided in the Supplementary Material. Participants on Prolific are nationals of or live in member countries of the Organisation for Economic Co-operation and Development (OECD) with the exception of Turkey, Lithuania, Colombia, and Costa Rica and the addition of South Africa (42).

### 2.5. Procedure

The study consisted of a questionnaire divided into two sections:

∙ *a pre-questionnaire section* to collect data on user characteristics, and
∙ *a scenario-rating section* in which participants were given 6 hypothetical scenarios with motivational messages intended to motivate the persona in the scenario per condition, for a total of 12 scenarios. To mitigate order effects and learning effects, ABBA counterbalancing was used when assigning tailored or generic messages. For each scenario, participants were asked to rate how motivating they would find the messages for themselves if they were the persona in the scenario. After the scenarios, participants were given two free-text questions about what they find motivating and demotivating in a motivational message.

### 2.6. Data preparation and analysis strategies

The data collected from the experiment was preprocessed by (1) removing data from participants whose submissions were not approved based on the earlier described criteria, and (2) anonymizing the data. The data and analysis script are available online (31) allowing to reproduce the analyses in a Docker container as recommended for Bayesian analyses by van de Schoot et al. (43).

We conducted a multi-level (i.e., hierarchical) Bayesian analysis using version 2.13 of the rethinking package (44). We fitted a model to a general mean, a random intercept for each participant, and a fixed effect for tailoring using diffuse priors based on the ones by McElreath (44).^1^ We fit a t-distribution to our output variable, which is the perceived motivational impact of the messages. Using a prior sensitivity analysis to assess the impact of different settings for the priors, we found that the posterior probability of our main hypothesis holding remained unchanged for the tested priors. Based on the fitted model, we computed the posterior probability that our hypothesis was true. More precisely, we calculated the posterior probability that the fixed effect for tailoring was greater than zero. This posterior probability was evaluated based on the guidelines by Chechile (38) and their extension to posterior probabilities of less than 0.5 by Andraszewicz et al. (45). We also report the 95% Highest Posterior Density Interval (HPDI) for estimators, with an HPDI being “the narrowest interval containing the specified probability mass” (44).

In addition, we performed a thematic analysis (46) of participants’ free-text responses about what they find motivating and demotivating about motivational messages for physical activity. After familiarizing herself with the data, RG created an initial coding scheme and coded all responses according to this scheme. This means that codes were largely created inductively. To assess the reliability of the coding, KP was trained on 20 responses for motivating factors and 10 responses for demotivating factors before independently coding all remaining responses based on the coding scheme. Afterward, the coding scheme was refined by RG and KP, and coding disagreements were resolved by means of discussion. The updated coding scheme was thus developed by multiple researchers, which has been described as a way to increase the validity of qualitative research (47). To evaluate the reliability of this updated coding scheme, we conducted a second round of double coding with NA. After being trained on 20 responses each for motivating and demotivating factors, NA coded all remaining responses. We obtained fair to moderate agreement (34) based on a Cohen’s κ of 0.40 for motivating and 0.46 for demotivating factors. In our analysis, we consider only those codes with at least moderate agreement (i.e., a Cohen’s κ of at least 0.41). For these codes that we consider in our analysis, the agreement is substantial to almost perfect based on a Cohen’s κ of 0.69 for motivating and 0.81 for demotivating factors. The final coding scheme together with the corresponding Cohen’s κ values can be found in the Supplementary Material.

## 3. Results

### 3.1. Perceived motivational impact of tailored vs. generic messages

**Figure 3:**
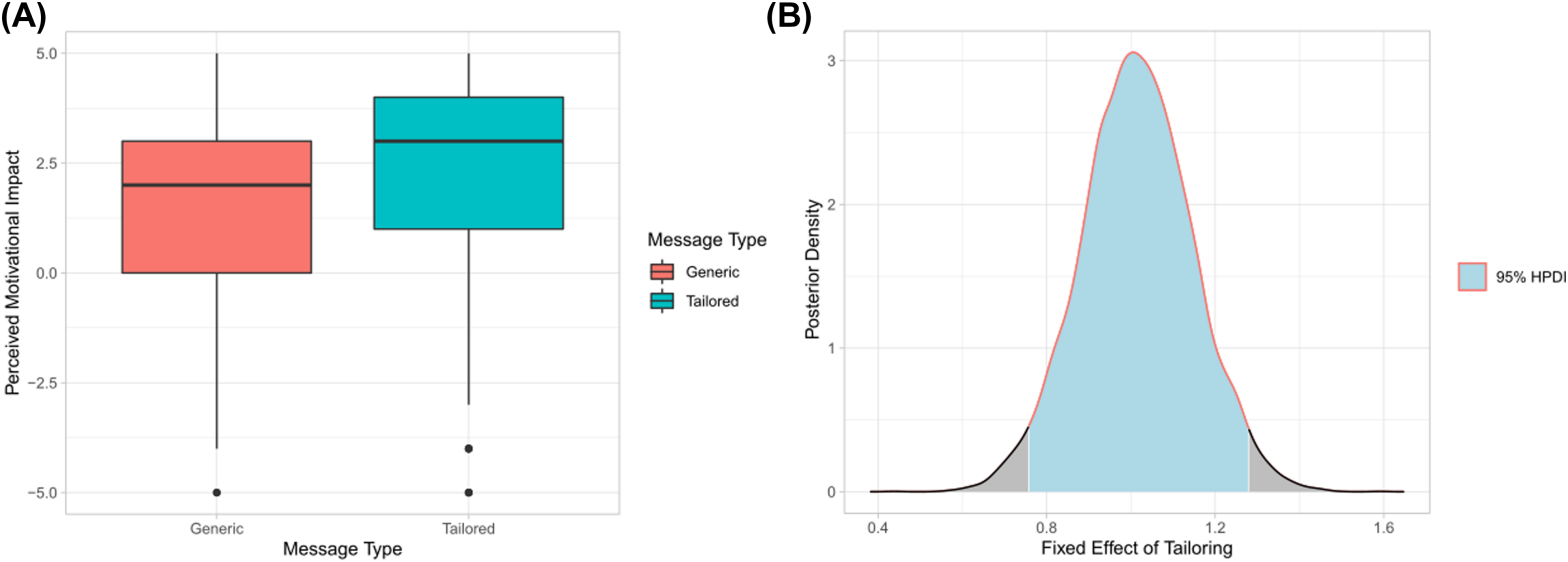
Perceived motivational impact of generic vs. tailored motivational messages. (**A**) Sample motivational impact ratings for the two types of messages. (**B**) Posterior density for the fixed effect of tailoring.

Figure 3A compares the sample motivational impact of the two message types. It shows that the sample mean perceived motivational impact is higher for tailored (M=2.33, SD=2.11) than for generic messages (M=1.32, SD=2.29). Quantifying this by means of our Bayesian analysis shows that the perceived motivational impact of tailored messages is 1.02 (SD=0.13) scale points higher than the one of generic messages. The corresponding 95%-HDPI ranges from 0.76 to 1.28, with >99.999% of the credibility mass favoring the higher motivational impact of tailored messages (Figure 3B). According to the guidelines by Chechile (38), this can be qualified, or can be “bet on,” as our hypothesis being virtually certainly true.

### 3.2. Exploratory analysis: motivating and demotivating factors of messages

**Figure 4:**
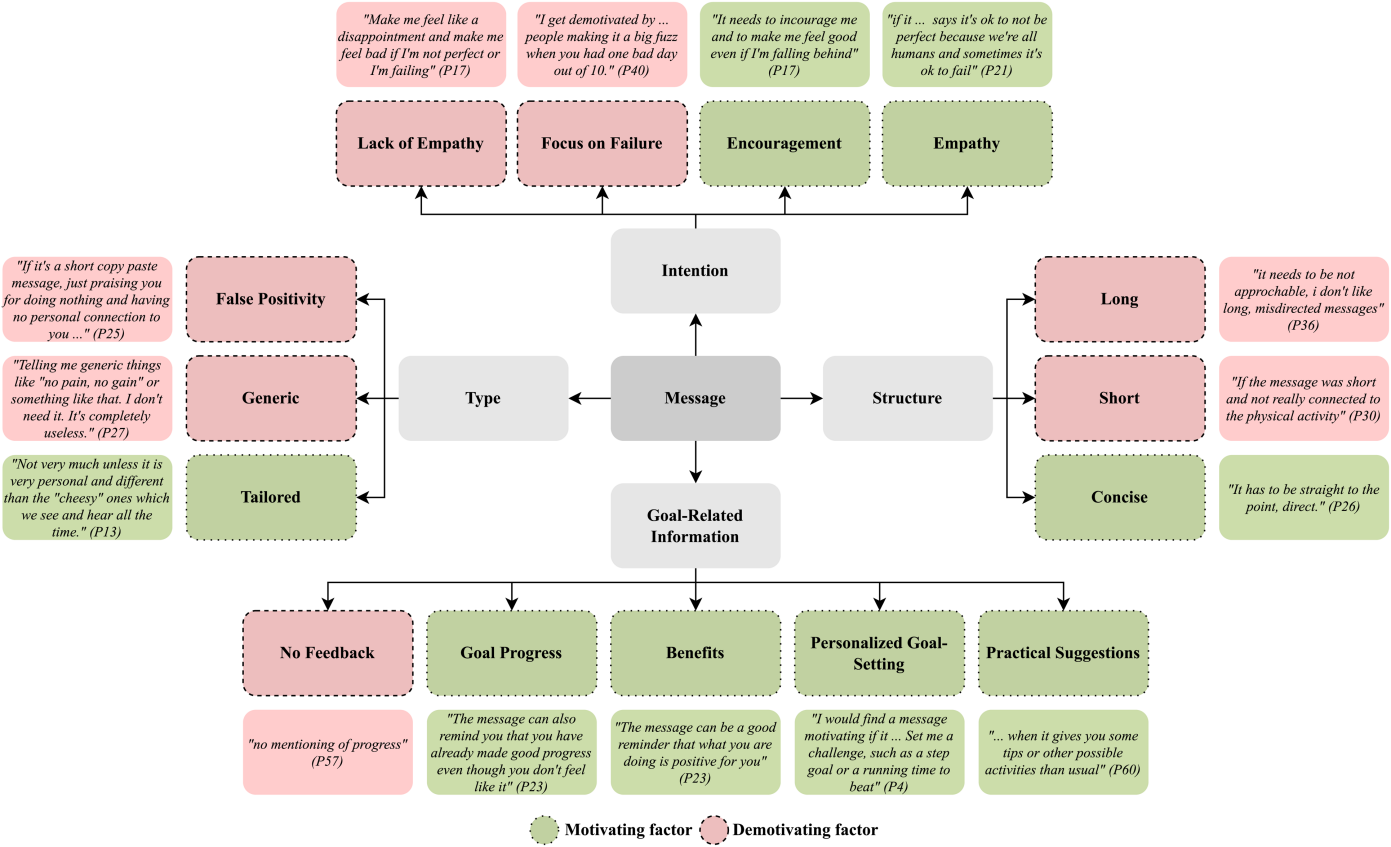
Themes describing factors participants found motivating and demotivating in a message, along with a few illustrative responses.

We identified four themes describing what participants found motivating and demotivating in a message (Figure 4).

**Tailored or generic.** The most frequent theme was whether a message is tailored to a particular user’s situation, or contains cliched, empty platitudes. Tailored messages were perceived as motivating, with 17% of the responses about motivating factors containing references to having personalized information in a message. On the other hand, messages with empty, cliched platitudes, or which conveyed a false sense of positivity, were reported to be demotivating. 40% of the responses about demotivating factors mentioned these attributes of generic messages. This is also demonstrated by the answer of P56 when asked about demotivating factors of a message: “If the message is generalized, I would rather not hear it at all and have an automated system, not a doctor, say it to me.” And when asked about motivating factors, P56 said: “If the message is specific to my own situation, so it seems more personal and tailored to myself, I would hear it with greater care and would adopt it easier.”

**Intention**. The intention of the message is the primary takeaway the message has. Encouraging and empathetic messages were the most commonly mentioned motivating factors here, appearing in 40% and 17% of the responses. On the other hand, messages that focus on failures or missed goals (22%) or lack empathy (10%) were seen as demotivating. P44 reported this when asked about what motivates them: “It must have some kind of encouragement for me, to make me understand that I can do it and I have what it takes.” Notably, however, too much empathy was also seen as demotivating by 7% of participants.

**Goal-related information**. Goal-related information encompasses all information related to a user’s physical activity goals. In motivating factors, the most commonly recurring sub-themes in goal-related information were receiving information on one’s progress toward a goal (20%), learning about the benefits of physical activity (17%) as well as alternatives for goals and ways to be physically active (17%), and setting personalized goals (10%). Conversely, a demotivating factor was a lack of feedback regarding goals (8%). P18, for example, reported this when asked about demotivating factors: “A message would demotivate me by not validating any progress made, not being understanding of my needs and not providing alternative solutions to reach my goals.”

**Message structure**. The structure of the message, specifically length and content style, was mentioned by a handful of participants. The responses about the message structure were conflicting, with short and long messages seen as demotivating in an equal percentage of responses (3%). For instance, P36 reported this when asked about demotivating factors: “…i don’t like long, misdirected messages.” P31, on the other hand, mentioned the following: “It is short and not personal. Completely random advice which does not help with my problem.” Overall it thus seems that participants agreed on not wanting generic, misdirected messages, and associated either short or long messages with this. This matches the observation that concise messages were seen as motivating.

## 4. Discussion

Based on 60 participants each rating the perceived motivational impact of six generic and six tailored messages based on scenarios, we find that messages tailored to a user’s mood, self-efficacy, and progress are perceived as more motivating than generic ones. This is in line with existing work which shows that tailored messages have modest success in motivating users in the context of health behavior change (1–4). Our results also complement existing research individually linking mood (48), self-efficacy (28), and progress (5) directly to motivation.

The thematic analysis of the motivating and demotivating factors of messages further revealed that motivation and de-motivation had common but complementary themes. For instance, goal-related information was a theme that was discovered in both motivating and demotivating factors, where participants found information about their progress and the benefits of physical activity motivating. A less-frequently mentioned factor was personalized goal-setting. On the other hand, participants found a lack of feedback demotivating. These results can be seen as validating the design of our messages, as the inclusion of progress and feedback was a key component of the design. Several of these themes have also been found in previous qualitative studies on motivation for physical activity. Kappen et al. (49), for instance, saw in the context of an eight-week physical activity intervention for older adults that people were motivated to do physical activity by “accomplishing a goal,” which included being inspired by the in-app progress reports as well as by hitting pedometer targets. And participants of the physical and psychological intervention for breast cancer survivors by Sebri et al. (50) were primarily motivated to engage in physical activity by the benefits of physical activity such as improving their physical well-being. The fact that not all of the themes from previous studies on motivation for physical activity (e.g., enjoying outdoors (49)) appeared in our study suggests, however, that what people find motivating in general and in motivational messages may not necessarily be the same.

Furthermore, participants highlighted that messages pertaining to their own situation (i.e., tailored messages) would be motivating, whereas cliched, generic messages would be demotivating. It stands to reason that motivating and demotivating factors are two sides of the same coin. The observation that tailored messages are perceived as motivating supports our quantitative findings as well as previous qualitative findings on motivation for physical activity. Albers et al. (51), for example, saw in the context of examples from other people shown in a goal-setting dialog for physical activity that examples from people that participants could relate to, and were thus perceived as tailored to the participant, were perceived as motivating.

### 4.1. Limitations

The main limitation of our study is that participants rated the perceived motivational impact of the messages for themselves if they were the personas in hypothetical scenarios instead of in real situations. To minimize the risk involved in the experiment such as the risk of injury, and due to the restrictions around Covid-19, we regarded the hypothetical scenarios as a good alternative to a real-world evaluation. Such scenarios have, for example, also successfully been used in studies by De Vries et al. (52) and Tielman et al. (13) to evaluate tailored motivational messages. Nevertheless, it is unclear how well our scenario-based findings generalize to other scenarios as well as users who are themselves in the situations described by the scenarios. To obtain a more accurate assessment of the messages’ motivational impact, the messages would ideally be shown to users when they have made similar progress in their physical activity, or have a similar mood or self-efficacy. Conducting such a study also with other users besides crowd workers may further enhance the generalizability of the findings.

Another limitation is that only the perceived motivational impact of the messages was measured, and not the impact of the messages on users’ physical activity. As stated in the COM-B model (30), motivation is only one of the three predictors of behavior, with opportunity and capability being the other two. Nevertheless, motivation has been shown to be a good predictor of behavior change (53–55), with the advantage of being easier to measure and being less noisy of a signal than actual behavior. Notably, even motivation can be talked about in terms of automatic vs. reflective according to the COM-B model (30). In our study, we considered solely reflective motivation, in which a user is actively and consciously involved in motivation, as opposed to automatic motivation, which is a result of impulsive, habitual, or drive-related behavior. However, the messages could also affect automatic motivation by, for example, influencing people’s self-identity (30).

Furthermore, our evaluation of the tailored motivational messages was based on comparing them to expert-written generic messages. Since the tailored messages tended to be more detailed than the generic ones, it could be that the higher perceived motivational impact of the tailored messages is partially due to their higher level of detail or larger number of characters. To rule this out, one could compare tailored messages that match scenarios to tailored messages that do not match scenarios. An example of the latter is a message written for a confident persona being evaluated on a scenario with a persona with low confidence. However, given that congratulating a person with low confidence for their high confidence is unlikely to be very motivating, we regarded generic messages as a stronger baseline.

### 4.2. Directions for future work

This research could be extended by automatically detecting a user’s state to tailor the messages and adapt content accordingly, as has been done based on affect by Grawemeyer et al. (56) in the context of student learning support. Similarly, Yang et al. (57) used sensors to automatically detect negative affect and send corresponding messages in the context of smoking cessation. To obtain more effective motivational messages, users’ responses to the messages could as a next step be recorded to learn what kinds of messages users prefer. Current work on reinforcement learning for determining the best time to send a message (58) and adapting the framing of messages for inducing healthy nutritional habits (59) makes the idea of tailoring messages by automatically adapting to user state variables a feasible next step.

Second, our ontology and resulting message structure can be used by developers of eHealth applications to automatically generate messages, similar to the work by Tielman et al. (13), Ghosh et al. (60) and Thomas et al. (14). For example, given a set of motivational messages like ours, new messages can be generated by combining the components from different messages. The fact that messages written based on the ontology can be broken down into components also makes it easier for future researchers to understand and reproduce our work. With the ontology given, messages can be obtained from crowd-workers as well, as demonstrated by De Vries et al. (52), making message generation cheap as well as time- and resource-efficient. Directly comparing different approaches to generating tailored motivational messages in terms of time and resource efficiency as well as effectiveness would also be worthwhile.

Thirdly, we only considered messages represented in text form. However, other types of representation such as images (61), videos (62), or even emoticons (63) are possible. For example, auditory feedback was used in a study by Singh et al. (64) to motivate people with chronic pain to be more physically active, and audio, visual, and haptic representations of motivational messages were proposed by Op den Akker et al. (27). Comparing how these various types of representation affect motivation and behavior is an interesting direction for future work. For instance, Symons et al. (65) found based on an exploratory study that people preferred GIFs over text reminders and pictures for being motivated to take a brisk walk in a difficult moment. As with the message content, the message representation could also be automatically adapted based on user feedback.

A fourth direction for future work is to tailor the messages to further factors besides mood, self-efficacy, and progress. In the context of persuasive messages more generally, both other dynamic factors (e.g., states derived from the COM-B model (17)) and more stable user characteristics (e.g., personality (18, 66), cultural background (67), regional factors (68), age (18)) have been shown to affect the effectiveness of different persuasive strategies. As the number of factors increases, so does the cost of collecting (expert-written or crowdsourced) messages tailored to these factors. However, not all of these factors are equally relevant. Albers et al. (20), for example, saw in the context of preparing for quitting smoking that dynamic factors could better predict people’s behavior after persuasive attempts than more stable user characteristics. First gaining a thorough understanding of which factors matter is thus important (20).

### 4.3. Conclusion

In conclusion, we provided a systematic theory-driven way to generate structured motivational messages with the help of experts that is feasible and can thus be used by developers of other eHealth applications for behavior change. Based on a scenario-based evaluation, we found credible support for our hypothesis that messages tailored to mood, self-efficacy, and progress are perceived as more motivating than generic messages in the context of physical activity coaching. Testing the combination of mood, self-efficacy, and progress as tailoring factors is completely new, to the best of our knowledge. A thematic analysis of people’s free-text responses about what they find motivating and demotivating about motivational messages provided further support for the use of tailored motivational messages, as well as messages that are encouraging and empathetic, give feedback about people’s progress and mention the benefits of physical activity. Our findings thus support the use of motivational messages tailored to mood, self-efficacy, and progress in eHealth applications for physical activity coaching. We share our dataset of motivational messages that can be used during various stages of a user’s physical activity intervention, along with a set of scenarios containing the aforementioned user state.

## Data Availability

All data and analysis code is publicly available on 4TU. Research Data (31).

## Footnotes

^1^We used normal priors with μ=0 and σ=10 for both the general mean and the fixed effect for tailoring. The full model specification can be found in the online repository (31).

## References

[1] Bol N Smit ES Lustria MLA. Tailored health communication: opportunities, challenges in the digital era. Digit Health. (2020) 6:2055207620958913. 10.1177/2055207620958913 33029355 PMC7520919

[2] Curry SJ. eHealth research, healthcare delivery: beyond intervention effectiveness. Am J Prev Med. (2007) 32:S127–30. 10.1016/j.amepre.2007.01.026 17466817

[3] Kreuter MW Wray RJ. Tailored, targeted health communication: strategies for enhancing information relevance. Am J Health Behav. (2003) 27:S227–32. 10.5993/AJHB.27.1.s3.6 14672383

[4] Smeets T Brug J de Vries H. Effects of tailoring health messages on physical activity. Health Educ Res. (2008) 23:402–13. 10.1093/her/cyl101 17032705

[5] Rutherford WJ Corbin CB Chase LA. Factors influencing intrinsic motivation towards physical activity. Health Values: The Journal of Health Behavior, Education & Promotion. (1992) 16(5):19–24.

[6] Figueroa CA Deliu N Chakraborty B Modiri A Xu J Aggarwal J, et al. Daily motivational text messages to promote physical activity in university students: results from a microrandomized trial. Ann Behav Med. (2022) 56:212–8. 10.1093/abm/kaab028 33871015

[7] Hors-Fraile S Schneider F Fernandez-Luque L Luna-Perejon F Civit A Spachos D, et al. Tailoring motivational health messages for smoking cessation using an mHealth recommender system integrated with an electronic health record: a study protocol. BMC Public Health. (2018) 18:1–10. 10.1186/s12889-018-5612-5 PMC5989385 29871595

[8] Hors-Fraile S Malwade S Luna-Perejon F Amaya C Civit A Schneider F, et al. Opening the black box: explaining the process of basing a health recommender system on the I-change behavioral change model. IEEE Access. (2019) 7:176525–40. 10.1109/ACCESS.2019.2957696

[9] Maurits K Ruyter Boris D Panos M Emile A. Adaptive persuasive systems: a Study of tailored persuasive text messages to reduce snacking. ACM Transactions on Interactive Intelligent Systems (TiiS). Vol. 2. New York, NY, USA: Association for Computing Machinery (2012). 10.1145/2209310.2209313

[10] Ybarra ML Prescott TL Holtrop JS. Steps in tailoring a text messaging–based smoking cessation program for young adults. J Health Commun. (2014) 19:1393–407. 10.1080/10810730.2014.901441 24766267

[11] Beristain Iraola A Álvarez Sánchez R Petsani D Hors-Fraile S Bamidis P Konstantinidis E. Virtual coaching for older adults at home using smart goal supported behavior change. Companion Publication of the 2020 International Conference on Multimodal Interaction. New York, NY, USA: Association for Computing Machinery (2020). p. 251–5. 10.1145/3395035.3425311

[12] Kang Y Tan AH Miao C. An adaptive computational model for personalized persuasion. *Proceedings of the 24th International Conference on Artificial Intelligence*. AAAI Press / International Joint Conferences on Artificial Intelligence (2015). p. 61–7.

[13] Tielman ML Neerincx MA Brinkman WP. Design, evaluation of personalized motivational messages by a virtual agent that assists in post-traumatic stress disorder therapy. J Med Internet Res. (2019) 21:e9240. 10.2196/jmir.9240 30916660 PMC6456821

[14] Thomas R Oren N Masthoff J. Argumessage: a system for automation of message generation using argumentation schemes. *Is ArguMessage Effective? A Critical Evaluation of the Persuasive Message Generation System* (2018). p. 27–31. https://link.springer.com/chapter/10.1007/978-3-030-17287-9_8

[15] Cauchard JR Frey J Zahrt O Krister J Alia C Landay JA. The positive impact of push vs pull progress feedback: a 6-week activity tracking study in the wild. *Proceedings of the ACM on Interactive, Mobile, Wearable, Ubiquitous Technologies*. New York, NY, USA: Association for Computing Machinery (2019) 10.1145/3351234.

[16] Bertolotti M Carfora V Catellani P. Different frames to reduce red meat intake: the moderating role of self-efficacy. Health Commun. (2019) 35:475–82. 10.1080/10410236.2019.1567444 30676108

[17] Albers N Neerincx MA Brinkman WP. Addressing people’s current and future states in a reinforcement learning algorithm for persuading to quit smoking and to be physically active. PLoS ONE. (2022) 17:e0277295. 10.1371/journal.pone.0277295 36454782 PMC9714722

[18] Kaptein M Eckles D. Heterogeneity in the effects of online persuasion. J Interact Mark. (2012) 26:176–88. 10.1016/j.intmar.2012.02.002

[19] Steward WT Schneider TR Pizarro J Salovey P. Need for cognition moderates responses to framed smoking-cessation messages. J Appl Soc Psychol. (2003) 33:2439–64. 10.1111/j.1559-1816.2003.tb02775.x

[20] Albers N Neerincx MA Brinkman WP. Persuading to prepare for quitting smoking with a virtual coach: using states and user characteristics to predict behavior. Proceedings of the 2023 International Conference on Autonomous Agents and Multiagent Systems. Richland, SC, USA: International Foundation for Autonomous Agents and Multiagent Systems (2023). p. 717–26.

[21] Bandura A Cervone D. Self-evaluative and self-efficacy mechanisms governing the motivational effects of goal systems. J Pers Soc Psychol. (1983) 45:1017. 10.1037/0022-3514.45.5.1017

[22] Dijkstra A. Working mechanisms of computer-tailored health education: evidence from smoking cessation. Health Educ Res. (2005) 20:527–39. 10.1093/her/cyh014 15701665

[23] Maheswaran D Meyers-Levy J. The influence of message framing and issue involvement. J Mark Res. (1990) 27:361–7. 10.1177/002224379002700310

[24] Petty RE Cacioppo JT. The elaboration likelihood model of persuasion. *Communication and persuasion*. Springer (1986). p. 1–24

[25] Bless H Bohner G Schwarz N Strack F. Mood and persuasion. Pers Soc Psychol Bull. (1990) 16:331–45. 10.1177/0146167290162013

[26] Biddle SJ Mutrie N Gorely T Faulkner G. Psychology of physical activity: determinants, well-being and interventions. New York: Routledge (2021).

[27] Op den Akker H Cabrita M Jones VM Hermens HJ. Tailored motivational message generation: a model, practical framework for real-time physical activity coaching. J Biomed Inform. (2015) 55:104–15. 10.1016/j.jbi.2015.03.005 25843359

[28] Riet J Ruiter RA Werrij MQ De Vries H. The influence of self-efficacy on the effects of framed health messages. Eur J Soc Psychol. (2008) 38:800–9. 10.1002/ejsp.496

[29] Hooker SA Masters KS. Daily meaning salience, physical activity in previously inactive exercise initiates. Health Psychol. (2018) 37:344. 10.1037/hea0000599 29369678

[30] Michie S Van Stralen MM West R. The behaviour change wheel: a new method for characterising and designing behaviour change interventions. Implement Sci. (2011) 6:1–12. 10.1186/1748-5908-6-42 21513547 PMC3096582

[31] Ghantasala R Albers N Penfornis KM van Vliet M Brinkman W-P. Feasibility of generating structured motivational messages for tailored physical activity coaching: data and analysis code. Version 1. 4TU. Research Data. (2023). 10.4121/33888406-2d4e-4365-bf6e-0a45616842ef.v1 PMC10523307 37771819

[32] Ghantasala RP Albers N. Motivational messages adapted to mood, self-efficacy, and progress (2021). Available from: 10.17605/OSF.IO/MT3FJ

[33] Stoet G Hommel B. Action planning and the temporal binding of response codes. J Exp Psychol Hum Percept Perform. (1999) 25:1625. 10.1037/0096-1523.25.6.1625

[34] Landis JR Koch GG. The measurement of observer agreement for categorical data. Biometrics. (1977) 6:159–74. 10.2307/2529310 843571

[35] de Vries RA Truong KP Zaga C Li J Evers V. A word of advice: how to tailor motivational text messages based on behavior change theory to personality and gender. Pers Ubiquitous Comput. (2017) 21:675–87. 10.1007/s00779-017-1025-1

[36] Fukuoka Y Lindgren TG Mintz YD Hooper J Aswani A. Applying natural language processing to understand motivational profiles for maintaining physical activity after a mobile app and accelerometer-based intervention: the mPED randomized controlled trial. JMIR Mhealth Uhealth. (2018) 6:e10042. 10.2196/10042 29925491 PMC6031900

[37] Cohen BH. Explaining psychological statistics. John Wiley & Sons (2008).

[38] Chechile RA. Bayesian statistics for experimental scientists. MIT Press (2020). p. 238.

[39] Schild C Lilleholt L Zettler I. Behavior in cheating paradigms is linked to overall approval rates of crowdworkers. J Behav Decis Mak. (2021) 34:157–66. 10.1002/bdm.2195

[40] Prolific Team. How do I set up a longitudinal/multi-part study? (2021). Available from: https://researcher-help.prolific.co/hc/en-gb/articles/360009222733-How-do-I-set-up-a-longitudinal-multi-part-study- (Accessed December 21, 2021).

[41] Draws T Rieger A Inel O Gadiraju U Tintarev N. A checklist to combat cognitive biases in crowdsourcing. *Proceedings of the AAAI Conference on Human Computation, Crowdsourcing*. Vol. 9. Palo Alto, California, USA: The AAAI Press (2021). p. 48–59. 10.1609/hcomp.v9i1.18939

[42] Prolific Team. Who are the participants on prolific? (2022). Available from: https://researcher-help.prolific.co/hc/en-gb/articles/360009220833-Who-are-the-participants-on-Prolific (Accessed June 20, 2022).

[43] van de Schoot R Depaoli S King R Kramer B Märtens K Tadesse MG, et al. Bayesian statistics, modelling. Nat Rev Methods Primers. (2021) 1:1–26. 10.1038/s43586-020-00001-2

[44] McElreath R. Statistical rethinking: a Bayesian course with examples in R and Stan. New York: Chapman and Hall/CRC (2020).

[45] Andraszewicz S Scheibehenne B Rieskamp J Grasman R Verhagen J Wagenmakers EJ. An introduction to Bayesian hypothesis testing for management research. J Manage. (2015) 41:521–43. 10.1177/0149206314560412

[46] Braun V Clarke V. Using thematic analysis in psychology. Qual Res Psychol. (2006) 3:77–101. 10.1191/1478088706qp063oa

[47] Nancy Carter R Bryant-Lukosius D Alba DiCenso R. The use of triangulation in qualitative research. *Oncology nursing forum*. Vol. 41. Oncology Nursing Society (2014). p. 545–7. 10.1188/14.ONF.545-547 25158659

[48] Bohner G Crow K Erb HP Schwarz N. Affect and persuasion: mood effects on the processing of message content and context cues and on subsequent behaviour. Eur J Soc Psychol. (1992) 22:511–30. 10.1002/ejsp.2420220602

[49] Kappen DL Mirza-Babaei P Nacke LE. Technology facilitates physical activity through gamification: a thematic analysis of an 8-week study. Front Computer Sci. (2020) 2:530309. 10.3389/fcomp.2020.530309

[50] Sebri V Durosini I Mazzoni D Pravettoni G. Breast cancer survivors’ motivation to participate in a tailored physical and psychological intervention: a qualitative thematic analysis. Behav Sci. (2022) 12:271. 10.3390/bs12080271 36004842 PMC9404874

[51] Albers N Hizli B Scheltinga BL Meijer E Brinkman WP. Setting physical activity goals with a virtual coach: vicarious experiences, personalization and acceptance. J Med Syst. (2023) 47:15. 10.1007/s10916-022-01899-9 36710276 PMC9884656

[52] De Vries RA Truong KP Kwint S Drossaert CH Evers V. Crowd-designed motivation: motivational messages for exercise adherence based on behavior change theory. *Proceedings of the 2016 CHI Conference on Human Factors in Computing Systems*. New York, NY, USA: Association for Computing Machinery (2016). p. 297–308. 10.1145/2858036.2858229

[53] Edmunds J Ntoumanis N Duda JL. A test of self-determination theory in the exercise domain. J Appl Soc Psychol. (2006) 36:2240–65. 10.1111/j.0021-9029.2006.00102.x

[54] Sheeran P Wright CE Avishai A Villegas ME Rothman AJ Klein WM. Does increasing autonomous motivation or perceived competence lead to health behavior change? A meta-analysis. Health Psychol. (2021) 40:706. 10.1037/hea0001111 34881939

[55] Sweet SN Fortier MS Strachan SM Blanchard CM. Testing and integrating self-determination theory and self-efficacy theory in a physical activity context. Can Psychol. (2012) 53:319. 10.1037/a0030280

[56] Grawemeyer B Mavrikis M Holmes W Gutierrez-Santos S Wiedmann M Rummel N. Affecting off-task behaviour: how affect-aware feedback can improve student learning. *Proceedings of the Sixth International Conference on Learning Analytics & knowledge*. New York, NY, USA: Association for Computing Machinery (2016). p. 104–13. 10.1145/2883851.2883936

[57] Yang MJ Sutton SK Hernandez LM Jones SR Wetter DW Kumar S, et al. A just-in-time adaptive intervention (JITAI) for smoking cessation: feasibility and acceptability findings. Addict Behav. (2023) 136:107467. 10.1016/j.addbeh.2022.107467 36037610 PMC10246550

[58] Liao P Greenewald K Klasnja P Murphy S. Personalized heartsteps: a reinforcement learning algorithm for optimizing physical activity. Proc ACM Interact Mob Wearable Ubiquitous Technol. (2020) 4:1–22. 10.1145/3381007 34527853 PMC8439432

[59] Carfora V Di Massimo F Rastelli R Catellani P Piastra M. Dialogue management in conversational agents through psychology of persuasion, and machine learning. Multimed Tools Appl. (2020) 79:35949–71. 10.1007/s11042-020-09178-w

[60] Ghosh S Chollet M Laksana E Morency LP Scherer S. Affect-LM: a neural language model for customizable affective text generation [Preprint] (2017). Available at: 10.48550/arXiv.1704.06851

[61] Wang W van Lint CL Brinkman WP Rövekamp TJ van Dijk S van der Boog P, et al. Guided or factual computer support for kidney patients with different experience levels and medical health situations: preferences and usage. Health Technol. (2019) 9:329–42. 10.1007/s12553-019-00295-7

[62] Whittaker R Dorey E Bramley D Bullen C Denny S Elley CR, et al. A theory-based video messaging mobile phone intervention for smoking cessation: randomized controlled trial. J Med Internet Res. (2011) 13:e1553. 10.2196/jmir.1553 PMC3221331 21371991

[63] Gill A Plasqui G Kok G Schols AM Ruiter RA Kremers SP, et al. Weight-status related differences in reflective and impulsive determinants of physical activity in youngsters (8–18 years old). Health Psychol Bull. (2020) 4:29–38. 10.5334/hpb.14

[64] Singh A Klapper A Jia J Fidalgo A Tajadura-Jiménez A Kanakam N, et al. Motivating people with chronic pain to do physical activity: opportunities for technology design. *Proceedings of the SIGCHI Conference on Human Factors in Computing Systems*. New York, NY, USA: Association for Computing Machinery (2014). p. 2803–12. 10.1145/2556288.2557268

[65] Symons M Vandebosch H Cutello CA Poels K. Message reminders encouraging brisk walking by considering the dynamic factor of cognitive fatigue. Eur J Health Commun. (2022) 3:41–68. 10.47368/ejhc.2022.303

[66] Zalake M de Siqueira AG Vaddiparti K Lok B. The effects of virtual human’s verbal persuasion strategies on user intention and behavior. Int J Hum Comput Stud. (2021) 156:102708. 10.1016/j.ijhcs.2021.102708

[67] Oyibo K Adaji I Orji R Olabenjo B Vassileva J. Susceptibility to persuasive strategies: a comparative analysis of Nigerians vs. Canadians. *Proceedings of the 26th Conference on User Modeling, Adaptation and Personalization*. New York, NY, USA: Association for Computing Machinery (2018). p. 229–38. 10.1145/3209219.3209239

[68] Xu W Legaspi R Ishikawa Y. Does the association between persuasive strategies and Personality types vary across regions. *Persuasive Technology: 18th International Conference, PERSUASIVE 2023, Proceedings*; 2023 Apr 19–21; Eindhoven, The Netherlands. Springer (2023). p. 359–68.

[69] Ghantasala R. *Motivating, your way: tailoring your fitness journey* [Master’s thesis] (2022). http://resolver.tudelft.nl/uuid:d2a3e006-5d75-404f-a0b9-89423ea97c63

