# Supplementary material for: Feasibility of generating structured motivational messages for tailored physical activity coaching
Source: Front Digit Health. 2023 Sep 12;5:1215187. doi: 10.3389/fdgth.2023.1215187 (PMC10523307; doi:10.3389/fdgth.2023.1215187)
Supplement: Supplementary file 1 [file Table1.pdf]

Supplementary Material

1 PARTICIPANT CHARACTERISTICS

Table S1. Characteristics of the 60 participants.

Characteristic	Value
**Age**	
Mean (SD)	28 (7)
Range	19 – 46
**Gender**	
Female, n (%)	24 (40%)
Male, n (%)	35 (58%)
Other, n (%)	1 (2%)
**Weekly Exercise Amount**	
Never (0 — 60 minutes per week), n (%)	10 (17%)
Sometimes (60 — 150 minutes per week), n (%)	35 (58%)
Often (more than 150 minutes per week), n (%)	15 (25%)
**Physical Activity Stage of Change (TTM)**	
Precontemplation, n (%)	2 (3%)
Contemplation, n (%)	12 (20%)
Preparation, n (%)	11 (18%)
Action, n (%)	16 (27%)
Maintenance, n (%)	19 (32%)

Abbreviations: SD, Standard deviation; TTM, Transtheoretical Model.

2 CODING SCHEME

Table S2. Coding scheme for demotivating factors with Cohen's κ values for each code. Codes with at least moderate agreement that are thus used in the analysis are shown in boldface.

Code	Definition	Cohen's κ
**Generic**	Generic, cliched message that has no personal relevance to the participant	0.85
**Focus On Failure**	Messages that focus on not achieving goals	0.49
Negative	Messages with a pessimistic or negative tone	0.21
**False Positivity**	Overly cheerful or messages with fake/false positivity	1.00
Negative Feedback	Negative criticism, or feedback that only deals with the negatives	0.18
**Social Comparison**	Comparing to others (even as an implication), especially an inferior comparison	1.00
**No Feedback**	Providing no feedback about progress made towards goals	0.55
**Authoritative**	Directive type messages that ”orders” the participant around	0.79
**Too Much Empathy**	Messages with too much empathy and understanding when the participant has not been reaching their goals	1.00
**Lack Of Empathy**	Little to no empathy or understanding when the participant is unable to reach their goals	0.55
**Short**	Messages with short message length, containing little to no useful information	1.00
Generic Goal Setting	Messages talking about goals that are not tailored to the participant, especially if they are generic or vague goals	0.00
Difficult Goals	Setting goals that are too difficult for the participant	-0.02
Verbose	Long messages that do not contain useful information but are instead filled with fluff	0.00
**Too Many Messages**	Receiving too many messages in a short span of time	1.00
Superficial	Messages that focus on the ”superficial” results of physical activity	0.38
Small Wins	Messages that constantly celebrate small achievements, especially if these achievements have been done many times before	0.00
**Pushing During Bad Mood**	Messages that actively disregard the state of mind of the participant, and only focus on achieving goals	0.49
Not Encouraging	Messages that are not encouraging the participant to do physical activity	0.00
**Non-Sensical**	Messages that plainly do not make any sense, or are not relevant at all	0.66
**No Suggestions**	Not providing alternative goals or physical activity suggestions	0.66
No Short-Term Wins	Focus only on the big picture and final goal, without celebrating any small wins along the way	-0.02
**No Reinforcement**	No positive reinforcement for the goals achieved	0.66
No Encouragement	Messages that do not encourage the participant	0.20
Misdirected	Messages full of fluff, and not actually about physical activity	0.00
**Long**	Length of the message is too long	1.00
Lack Of Self-Efficacy	Difficult goals that specifically make the participant think they lack the self-efficacy to achieve the goal	0.00
**Information**	Too much information about physical activity and its side-effects	1.00
Focus On Achievements	Focus only on achieving goals, for instance, regardless of whether the goals are healthy or not	0.00
Easy Goals	Goals that are too easy or are not challenging enough	0.00
**Daily Reminders**	Getting reminded daily to do physical activity or achieve their goals	1.00
Patronizing	Downsizing efforts, speaking to someone like they or their efforts are not important	0.15

Table S3. Coding scheme for motivating factors with Cohen's κ values for each code. Codes with at least moderate agreement that are thus used in the analysis are shown in boldface.

Code	Definition	Cohen's κ
**Encouragement**	Messages that encourage or motivate the participant to achieve their goals	0.41
**Goal Progress**	Mentioning the participant's progress towards their goal, feedback about their progress	0.43
**Benefits**	Talking about the benefits of physical activity	0.84
**Empathy**	Being empathetic and understanding when the participant has not achieved their goals	0.42
**Tailored**	Messages that are tailored or personalized, are not generic, and do not contain cliches or empty platitudes	0.68
**Personalized Goal Setting**	Setting specific goals tailored to the individual instead of vague, generic ones	0.57
Uplifting	Messages that have a positive tone, makes the participant feel "nice" and cheered on	0.30
**Positive Reinforcement**	Positively reinforcing the participant's progress by celebrating their progress and achievements	0.53
**Practical Suggestions**	Providing practical alternatives for goals or ways to be physically active	0.68
**Small Wins**	Messages that keep track of milestones, and goals achieved while showing visible signs of progress	0.66
Short	Messages that are too short in length, and do not contain the necessary information	0.00
Reminder Of Motivation Behind Behavior Change	Messages that remind the person of the initial reason that motivated them to be physically active	0.39
**Concise**	Messages that are concise and do not contain fluff or empty words	1.00
Trust Person	Messages that are sent by a trusted person	0.00
Support	Messages that reassure the person, especially with regards to their self-efficacy	-0.03
**Social Proof**	Messages that encourage a person to be physically active because other people are being physically active	0.66
**Social Competition**	Messages that encourage a person to be physically active by competing with other people who are physically active	1.00
Simple	Content of the message is clear and well-written	0.00
Sensible	Messages that make sense, and are about physical activity	0.00
**Rewards**	Participant finds it motivating when they are rewarded for making progress	1.00
Reminders	Frequent reminders about goals the participant has set	-0.01
Past Experiences With Pa	Reminding the participant about their past experience with physical activity, and the success they've had	0.00
Intrinsic Motivation	Participant finds it motivating when they are already prepared to be physically active, i.e., they are intrinsically motivated	0.00
Effective	Participant finds messages to be motivating when they are effectively shown to be improving physical activity	0.00
Easy-Going	Messages that do not drill or pressurize the participant to work out every day	0.00
Community	Messages that provide a sense of community to the participant so they don't feel alone	0.38
**Big Picture**	Messages that focus on the big picture and not stress over the small things (failures)	0.79
